# Gray Wolves Help Scavengers Ride Out Climate Change

**DOI:** 10.1371/journal.pbio.0030132

**Published:** 2005-03-15

Average earth temperatures rose 0.6 °C over the last century, according to the latest Intergovernmental Panel on Climate Change. But that increase pales in comparison to the 1.4–5.8 °C expected increase over this century. As temperatures climb, climate models predict that high-latitude, high-altitude regions like Yellowstone National Park will experience shorter winters and earlier snow melts. How these environmental shifts will impact species and ecosystems remains to be seen.

The effects of climate change are already evident at the species level, with disruptions in range, reproductive success, and seasonal phenomena like migration, and the decoupling of evolutionarily paired events like new births and food availability. Both experimental and data-driven modeling studies predict that climate change may well precipitate shifts in the structure of ecosystems as well.

In a new study, Christopher Wilmers and Wayne Getz investigated the effects of climate change on ecosystem dynamics by studying a keystone species in Yellowstone, the gray wolf (Canis lupus). Gray wolves inhabited most of North America until US extirpation campaigns nearly eradicated them by the 1930s. In 1995, the US Fish and Wildlife Service reintroduced the persecuted predator into Yellowstone.

Wilmers and Getz used data from the past 50 years at two weather stations in the park's northern range (where elk over winter and four to six wolf packs now live) to establish winter trends and model wolves' impact on the fate of resident scavengers faced with a changing climate. Not surprisingly, their models show that this top predator exerts significant influence over animals at lower levels in the food chain: wolf kills temper the potentially devastating effects of climate-related carrion shortages on scavengers. Unlike mountain lions and grizzly bears, wolves abandon their prey (usually elk or moose) once sated, leaving much-coveted leftovers for ravens, eagles, coyotes, bears, and other scavengers. These findings indicate that individual species stand a better chance of adapting to climate change in an ecosystem with an intact food chain.

Wilmers and Getz's weather data analysis found that both late-winter snow depth and snow-cover duration have decreased significantly since 1948—winters in Yellowstone are getting shorter. That's good news for elk—navigating deep snow taxes stamina and reduces access to forage—but bad news for scavengers that rely on elk carcasses to carry them through the winter.

The authors generated two sets of models to estimate the effects of shorter winters on the wolf–elk–scavenger dynamics. In the first, late-winter carrion availability drops by 66% without wolves but by only 11% when the predators are present. The second model examines the impact of elk and wolf population dynamics on carrion availability. This analysis predicts that more elk will die in early winter than in late winter, a scenario that favors eagles and ravens—which can cover a lot of ground quickly—over bears and coyotes. Altogether, these modeling studies show that shorter winters without wolves will create intermittent food supplies that no longer track the needs of local scavengers. With or without wolves, late-winter carrion abundance will decline with shorter winters. But wolf kills buffer these shortages, providing meals that could determine whether scavengers will be able to survive and reproduce.[Fig pbio-0030132-g001]

**Figure pbio-0030132-g001:**
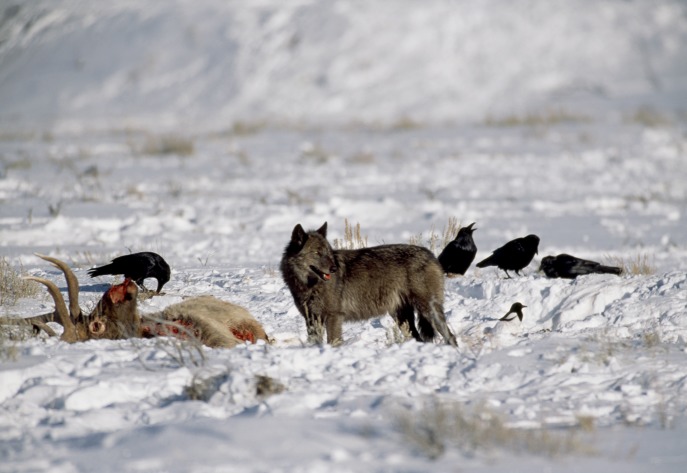
Reintroduced wolves do their part: an intact food chain buffers the impact of deteriorating environmental conditions (Photo: Dan Hartman)

It seems clear that wolves have the potential to provide a safety net for scavengers, extending the time they need to adapt to a changing environment. Thanks to a rebounding wolf population, field researchers can measure the magnitude of this predicted buffer effect. The models described here can guide their efforts and help species adjust to major environmental shifts like climate change.

As a young US ranger “full of trigger-itch,” Aldo Leopold killed his share of wolves under the federal eradication policy—until he “watched a fierce green fire dying” in the eyes of a slain mother flush with pups and realized he had not understood the wolf's ecological role. Wilmers and Getz's study shows that a robust food chain—including this still embattled top predator—may be even more important as ecological conditions deteriorate.

